# Biological, as opposed to classic antipsoriatic drug or apremilast, treatment mitigates the risk of death and cardiovascular disease in psoriasis

**DOI:** 10.1016/j.ebiom.2024.105485

**Published:** 2024-12-06

**Authors:** Khalaf Kridin, Katja Bieber, Artem Vorobyev, Eva Lotta Moderegger, Henning Olbrich, Marlene A. Ludwig, Bernard Gershater, Gema Hernandez, Henner Zirpel, Diamant Thaci, Ralf J. Ludwig

**Affiliations:** a Lübeck Institute of Experimental Dermatology, University of Lübeck, Lübeck, Germany; b Azrieli Faculty of Medicine, Bar-Ilan University, Safed, Israel; c Unit of Dermatology and Skin Research Laboratory, Galilee Medical Center, Nahariya, Israel; d Department of Dermatology, University Hospital Schleswig-Holstein Lübeck, Lübeck, Germany; e Independent Researcher, Groß Grönau, Germany; f Institute and Comprehensive Centre for Inflammation Medicine, University-Hospital Schleswig-Holstein, Lübeck, Germany; g TriNetX, LLC, Cambridge, MA, USA; h Biomedical Informatics Group, Artificial Intelligence Department, E.T.S.I. Informáticos, Universidad Politécnica de Madrid, Spain

**Keywords:** Psoriasis, Mortality, Major adverse cardiac events (MACE), Comorbidity, Cardiovascular, TriNetX

## Abstract

**Background:**

Cardiovascular comorbidity increases morbidity and mortality in psoriasis. Systemic treatments, particularly biologics, are effective in alleviating skin and joint inflammation. Conversely, the impact of systemic therapy on cardiovascular disease risk and mortality in psoriasis remains uncertain.

**Methods:**

Impact of systemic treatments on all-cause mortality and cardiovascular disease risk in psoriasis patients' electronic health records (EHRs) from TriNetX was assessed. Treatment categories included apremilast, IL-17 inhibitors (IL-17i), IL23i, TNFi, and classic antipsoriatic drugs. Index event was the first prescription of each treatment, requiring a two-year continuous treatment with exclusion of other systemic antipsoriatic drugs. Propensity-score matching was used to improve comparability. Sensitivity analyses ensured study robustness.

**Findings:**

In descriptive analysis, all-cause mortality rates were 0.61% (classic antipsoriatics, n = 7929), 0.91% (apremilast, n = 1101), 0.00% (IL17i, n = 677), 0.81% (IL23i, n = 1242), and 0.20% (TNFi, n = 6468). Major adverse cardiac events (MACE) were documented in 8.49% (classic antipsoriatics), 5.14% (apremilast), 2.99% (IL17i), 2.09% (IL23i), and 3.74% (TNFi) EHRs. Propensity-score matching showed all-cause mortality rates of 0.23% for any biologic vs. 0.49% for classic antipsoriatics or apremilast, resulting in an HR of 2.21 (95% CI 1.21–3.71, p = 0.0073). MACE risk was also higher with classic antipsoriatics or apremilast (HR 1.66, CI 1.43–1.93, p < 0.0001). The majority of findings were consistent across all four sensitivity analyses. No significant differences in all-cause mortality or MACE risk were observed among biologics.

**Interpretation:**

Biological treatment, as opposed to classic antipsoriatic drugs or apremilast, reduces risk of death and cardiovascular disease in psoriasis. Prospective trials are required to validate these findings.

**Funding:**

DFG: EXC 2167 and LU 877/25-1. State of Schleswig Holstein: Excellence-Chair Program.

**Research in context**

**Evidence before this study**

Individuals with psoriasis, a chronic inflammatory disease mainly affecting the skin and/or joints, are burdened by an elevated risk of cardiovascular disease and an increased likelihood of mortality. While systemic treatments, particularly biologics, effectively alleviate skin and joint inflammation, their impact on cardiovascular disease risk and all-cause mortality in psoriasis remained uncertain. Existing studies provided limited clarity on the comparative effects of different systemic therapies, leaving a gap in understanding the overall cardiovascular outcomes associated with these treatments.

**Added value of this study**

This study addressed the impact of different systemic treatments on all-cause mortality and cardiovascular disease risk in psoriasis patients. The use of electronic health records (EHRs) allowed for a comprehensive evaluation across various treatment categories, including apremilast, IL-17 inhibitors (IL-17i), IL23i, TNFi, and classic antipsoriatic drugs. The findings demonstrate distinct risks in terms of all-cause mortality rates and major adverse cardiac events (MACE) associated with each treatment. A comparative analysis between non-biologic and biologic antipsoriatic drugs highlights a notable reduction in risk in the latter group.

**Implications of all the available evidence**

These findings underscore the potential benefits of biological over non-biological treatment (classic antipsoriatic drugs or apremilast) in reducing the risk of death and cardiovascular disease in individuals with psoriasis. As these findings are based on retrospective analysis of EHRs, prospective trials are required for validation. In the interim, the observed decrease in risks for all-cause mortality and cardiovascular disease among patients treated with biologics supports the preference for choosing these over non-biologics as a favorable systemic first-line therapy for psoriasis.

## Introduction

Psoriasis, a chronic non-communicable disease, can affect the skin, nails, and/or joints, potentially causing itch, pain, disfigurement, and disability. Currently, causative treatment or cure is not possible.^1^^,^^2^ Despite significant advancements in psoriasis treatment, the disease remains associated with an elevated risk of (co)morbidity and increased mortality.^3^^,^^4^ Psoriasis has long been considered to be associated with cardiovascular disease.^5^ This presumption has been affirmed in the majority of epidemiological studies on the subject,^6^^,^^7^ and is further supported by genetic,^8^ biomarker^9^^,^^10^ and experimental studies.^11^ Therefore, cardiovascular disease significantly contributes to the morbidity and mortality of individuals with psoriasis.^12^

Biologic agents unquestionably led to groundbreaking improvement in patient care, greatly improving disease burden.^2^ By contrast, their effect on cardiovascular outcomes in psoriasis remains uncertain.^13^, ^14^, ^15^ Some studies suggested a protective role for biologics against the development of cardiovascular diseases,^16^, ^17^, ^18^, ^19^, ^20^ while other lines of evidence contradicted such a cardioprotective effect.^21^, ^22^, ^23^ Of note, several of these studies focused on biomarkers rather than cardiovascular disease or compared effects of individuals exposed to biological or topical treatment. Few studies considered cardiovascular disease risk factors as potential confounders. Consequently, bias stemming from the selection of biomarkers, variations in disease severity and an imbalanced distribution of cardiovascular risk factors may confound the results. A meta-analysis pooling 38 randomized controlled trials contrasting MACE risk between psoriasis patients treated with biologics as opposed to placebo or methotrexate detected no significant influence of biologics on the incidence of MACE.^24^ A potential confounding factor in this study is the relative low number of observed MACE. In detail, MACE was documented in 10 of 18,024 patients. Consistent with these findings, a recent retrospective cohort involving 10,782 psoriasis patients treated with either IL17i or IL23i observed a “sufficient” number of cardiovascular events (i.e., myocardial infarction in 0.9%) with no discernible difference between the two treatment groups.^25^ Hence, the impact of systemic antipsoriatic drugs, encompassing classic antipsoriatics (methotrexate, cyclosporine, acitretin, dimethyl fumarate), small molecules (apremilast) and biologics (TNFi, IL17i, IL23i), on the risk of mortality and cardiovascular disease remains largely elusive.

Yet, understanding the effects of systemic medications for psoriasis beyond skin and joint inflammation would be valuable in guiding treatment decisions. A recent retrospective cohort study emphasized this aspect in terms of the risk for psoriasis patients to develop inflammatory arthritis. The findings indicated that treatment with IL (12)/23i was associated with a reduced risk of subsequent inflammatory arthritis compared to TNFi.^26^ Pending validation through analysis of observational cohorts and/or meta-analysis of completed clinical trials, these results suggest a preference for IL (12)/23i over TNFi as the first-line biologic treatment for psoriasis. To extend these clinically relevant findings to mortality and cardiovascular diseases, the study presented herein contrasted and compared the risk of death and cardiovascular disease among patients treated with systemic antipsoriatic drugs, encompassing classic antipsoriatic drugs, apremilast, and biologics. To achieve this, a retrospective cohort study was performed in a federated network of real-world data. Examining the risk for cardiovascular disease in psoriasis is of significant merit, given that cardiovascular issues constitute a major cause of death among patients with psoriasis.^27^, ^28^, ^29^ In line, cardiovascular comorbidities have been identified as contributors to the excess mortality in psoriasis patients.^28^

## Methods

### Study design and database

**Fig. 1:**
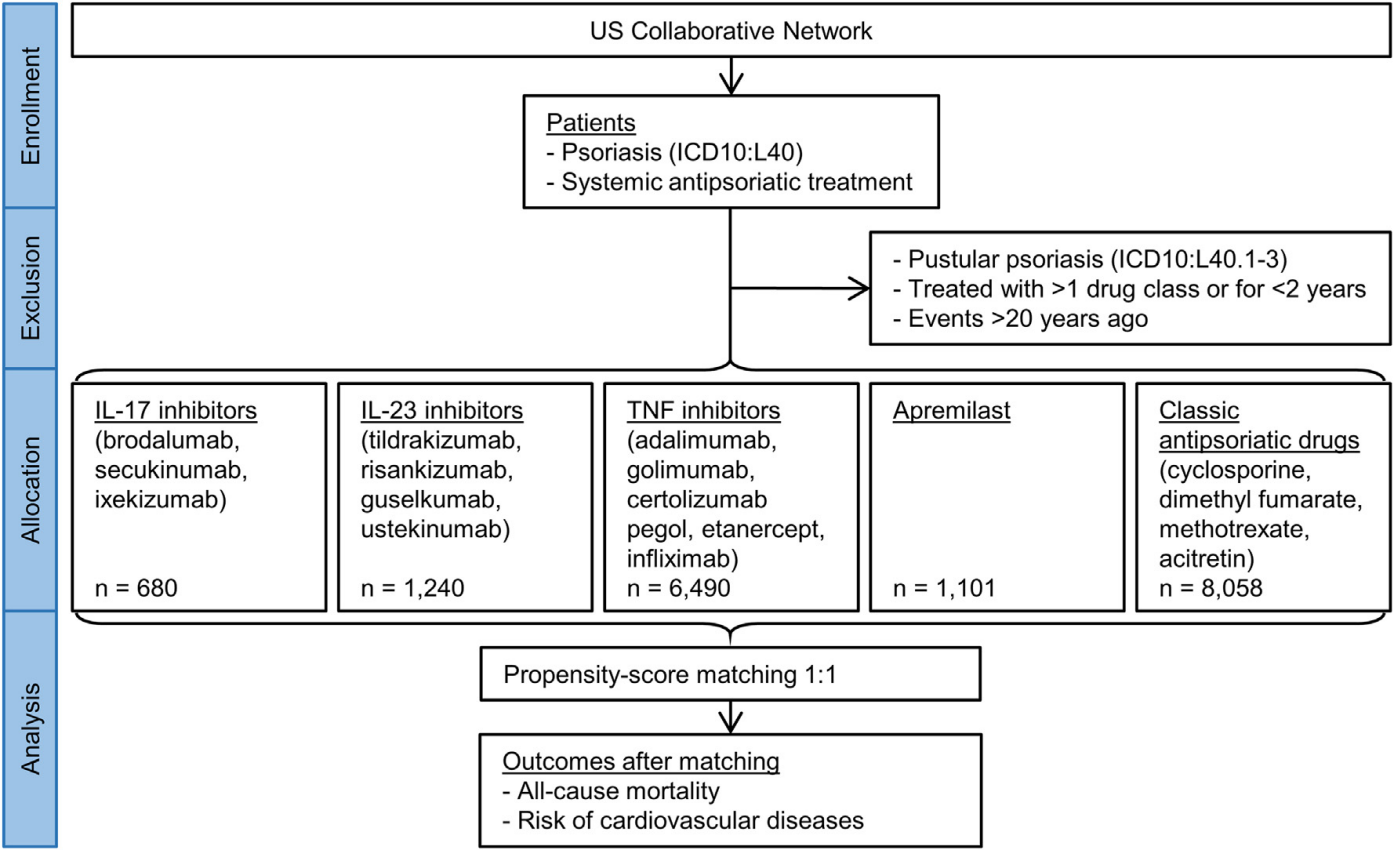
Flow chart of study design.

A global population-based retrospective cohort study with propensity-score matching was performed following previously published protocols.^30^, ^31^, ^32^ Electronic health records (EHRs) were sourced from the US Collaborative Network of TriNetX, a federated network of real-world data.^33^ As part of a collaboration between the University Clinic of Schleswig–Holstein (UKSH) and TriNetX, UKSH researchers have access to TriNetX. Cohorts of psoriasis patients under different systemic treatments were retrieved from the database. Treatments included apremilast, interleukin (IL)17 inhibitors (IL-17i), IL23i, TNF inhibitors, and classic antipsoriatic drugs. The index event was the first prescription of each treatment, necessitating a two-year continuous treatment period with exclusion of any other systemic antipsoriatic drugs. With this data, the following analyses were performed: (**i**) Description of the risk of death and cardiovascular disease in each group: For each treatment group, demographic information, cardiovascular risk factors, comorbidity, laboratory results, mortality, and the incidence of major adverse cardiac events (MACE) was assessed. Specific cardiovascular outcomes included heart failure, cardiac arrest, acute myocardial infarction, cerebral infarction, haemorrhagic stroke, peripheral arterial disease, thrombosis, and pulmonary embolism. (**ii**) In the primary comparative analysis, the risk of death and the aforementioned cardiovascular diseases between psoriasis patients exposed to biological treatments (TNFi, IL17i, or IL23i) and those exposed to non-biological treatments (classic antipsoriatics or apremilast) was determined. Propensity-score matching was employed to mitigate potential bias arising from unequal demographic or cardiovascular risk factor distributions. (**iii**) Lastly, four sensitivity analyses were performed to challenge validity of the study. The study flow chart summarizing the study design is shown in Fig. 1.

### Ethics statement

This retrospective study is exempt from informed consent. The data reviewed is a secondary analysis of existing data, does not involve intervention or interaction with human subjects, and is de-identified per the de-identification standard defined in Section §164.514(a) of the HIPAA Privacy Rule. The process by which the data is de-identified is attested to through a formal determination by a qualified expert as defined in Section §164.514(b) (1) of the HIPAA Privacy Rule. This formal determination by a qualified expert refreshed on December 2020.

### Study population and definition of eligible patients

EHRs of cases and controls were retrieved from the database based on ICD10 codes. For the primary analysis, psoriasis was defined by ICD10:L40, excluding all forms of pustular psoriasis (ICD10:L40.1-3). The treatment groups were defined using the corresponding RxNorm codes: Cyclosporine (RXNORM:3008), dimethyl fumarate (RXNORM:1373478), methotrexate (RXNORM:6851), or acitretin (RXNORM:16818) characterized the classic antipsoriatic group. The apremilast group was defined using apremilast (RXNORM:1492727). The IL17i group encompassed brodalumab (RXNORM:1872251), secukinumab (RXNORM:1599788), and ixekizumab (RXNORM:1745099). For the IL23i group, treatments included tildrakizumab (RXNORM:2053436), risankizumab (RXNORM:2166040), guselkumab (RXNORM: 1928589), or ustekinumab (RXNORM:847083). The TNFi group was defined by the use of adalimumab (RXNORM:327361), golimumab (RXNORM:819300), certolizumab pegol (RXNORM:709271), etanercept (RXNORM:214555), or infliximab (RXNORM:191831). Within each treatment group, the inclusion criteria stipulated the exclusion of any drugs from alternative categories. The index event was defined as the initiation of the first prescription for any given drug within the specified treatment category. To guarantee sustained drug exposure and facilitate follow-up, documentation of exposure was mandated at the index event and at any time 2 years thereafter. Same groups were used for the first two sensitivity analysis, whilst for the 3rd sensitivity analysis, continued drug exposure and follow-up was limited to one year. For the second sensitivity analysis, psoriasis was defined more leniently, including all EHRs with ICD10:L40.

### Covariates

In the initial analysis, demographic information such as age at the index, female sex, and White ethnicity, along with cardiovascular risk factors including family history of ischemic heart disease, current or past nicotine dependence, essential (primary) hypertension, disorders of lipoprotein metabolism, diabetes mellitus, and body mass index (BMI), were descriptively retrieved. Comorbidity data encompassed chronic lower respiratory diseases, chronic kidney disease, and neoplasms. Additionally, levels of C-reactive protein (CRP) were included in the descriptive retrieval. Alongside, the risk of death and cardiovascular disease was determined. When contrasting the risk of death and cardiovascular disease between differently treated groups propensity score matching including all these variables was employed. In detail, a propensity score for each patient was generated by logistic regression using the Python package “scikit-learn”. Matching was performed 1:1 using the greedy nearest neighbor approach with a cut-off distance of 0.1 pooled standard deviations of the logit of the propensity-score. Baseline characteristics were re-evaluated and reported after matching, while differences were compared by t-test for continuous variables and z-test for binary or categorical variables.

### Outcomes

Outcomes were defined by ICD10 codes: Myocardial infarction (ICD10:21-23), cerebral infarction (ICD10:I63), thrombosis (ICD10:I80, I82), and pulmonary embolism (ICD10:I26). ICD10:I21-23, I46, I50, I63 or R57.0 defined MACE. In the descriptive analysis, outcomes were evaluated from one day to two years following the index event. The primary comparative analysis considered outcomes during the same timeframe. In the first sensitivity analysis the observation period was limited to one month after the index event until two years thereafter. The second sensitivity analysis mirrored the follow-up period of the primary analysis. In the third sensitivity analysis, outcomes from one day to one year after the index event were considered. EHRs indicating outcomes (except death) prior to the index event were excluded from the analysis. A different approach was taken in the fourth sensitivity analysis, where any outcome occurring any time before or at the index event were excluded when retrieving the data, and follow-up time was for five years to ensure a sufficient number of outcomes. This analysis thus documents the first instance of the outcomes, whilst providing a more matched cohort. The date of analysis is indicated at the table and figure legends.

### Statistical analysis

Statistical analysis was performed on the TriNetX platform. Relative risks and risk differences were calculated. Survival analyses were performed using the Kaplan–Meier method (KM). KM-curves were compared using the Log-rank test; p values of less than 0.05 were considered significant. Hazard ratio and its associated confidence intervals, together with the test for proportionality, are calculated using R's Survival package v3.2–3, whereby large Chi-square values are indicative of low proportionality, whereas small Chi-square values suggest higher proportionality. To counter the bias of multiple testing, Bonferroni correction was employed, with α(adjust) = 0.008. Nelson–Aalen plots were utilized to test the proportionality assumption. A univariate Cox proportional hazards regression was used to express hazard ratios (HRs).

### Declaration of generative AI and AI-assisted technologies in the writing process

During the preparation of this work the authors used ChatGPT 3.5 (https://chat.openai.com) to edit the text and improve wording. After using ChatGPT, the author reviewed and edited the content as needed and take full responsibility for the content of the publication.

### Role of the funding source

The funders had no involvement in the design, execution, analysis, or interpretation of the study, nor in the writing or decision to submit the manuscript for publication.

## Results

### Descriptive analysis

In the descriptive analysis, a dataset comprising 17,569 individuals diagnosed with psoriasis and exposed to specific systemic antipsoriatic treatments for a minimum duration of two years was retrieved from the US Collaborative Network on December 17th, 2023. Among these, 8058 received traditional antipsoriatic medications, namely methotrexate, dimethyl fumarate, cyclosporine, or acitretin. The second-most applied treatment of 6490 EHRs, were TNFi. Other antipsoriatic treatments were observed with lower frequencies. IL23i were recorded in 1240 EHRs, with a reduced count of 312 when the IL12/23i ustekinumab was excluded. Apremilast was documented in 1101 patients, while IL17i were employed in 680 individuals (Table 1).

**Table 1:** Demographics, comorbidity and laboratory of the study population in the US collaborative network.

Characteristics/treatment	TNFi	IL17i	IL23i	IL23i, excluding ustekinumab	Apremilast	Classic
N	6490	680	1240	312	1101	8058
Age, years mean ± SD	46 ± 16.3	48.4 ± 15	46.1 ± 15.7	46.3 ± 14.2	54.2 ± 14	56.9 ± 16.5
Sex, female (%)	49	52	48	46	58	68
White (%)	81	79	80	78	82	83
Hispanic or Latino (%)	5	4	4	5	3	6
Black or African American (%)	6	5	5	5	3	8
Asian (%)	2	4	3	3	3	2
**Family history and comorbidity**						
Family history of ischemic heart disease and other diseases of the circulatory system (%)	3	5	3	5	5	7
Nicotine dependence (%)	7	9	8	13	8	9
Personal history of nicotine dependence (%)	6	7	7	8	11	9
Essential (primary) hypertension (%)	19	26	21	27	34	33
Disorders of lipoprotein metabolism and other lipidemias (%)	16	20	18	21	29	28
Chronic lower respiratory diseases (%)	10	9	10	14	16	26
Chronic kidney disease (%)	4	3	5	6	6	7
Neoplasms (%)	14	17	18	22	29	27
Diabetes mellitus (%)	9	13	9	10	17	14
**Vitals and laboratory**						
Body mass index, kg/m^2^ (mean [standard deviation])	28.9 ± 7.31	31.2 ± 7.63	30.7 ± 7.2	32.6 ± 7.23	30.5 ± 6.27	29.7 ± 7.2
C reactive protein, mg/L (mean [standard deviation])	17.6 ± 33	8.87 ± 13.9	10.6 ± 16.4	6.81 ± 13.5	12 ± 25.7	15 ± 30.9

Analysis performed on December 17th, 2023.

Before propensity-score matching, significant disparities were observed in the demographic characteristics. The mean age (SD) ranged from 46 ± 16.3 years, among psoriasis patients exposed to TNFi, to 56.9 ± 16.5 years in those prescribed classic antipsoriatic drugs. The IL23i had the lowest proportion of female EHRs at 48%, while the classic antipsoriatic drugs group reported a higher percentage at 68%. By contrast, ethnic diversity demonstrated relatively uniform distribution across all treatment cohorts (Table 1). Risk factors of cardiovascular disease,^34^ comorbidity and C reactive protein (CRP) serum also showed a high degree of variability among the groups. In detail, the lowest proportion of a “family history of ischemic heart disease” was seen in IL23i treated psoriasis patients. In those treated with classic antipsoriatic drugs, this amounted to 7%. Prevalence of essential hypertension was again lowest in the IL23i group (21%) and highest in the classic antipsoriatic group (33%). Body mass index (BMI, mean [SD]) varied from 28.9 ± 7.31 kg/m^2^ (TNFi) to 31.2 ± 7.63 kg/m^2^ (IL17i). CRP concentrations varied from 8.87 ± 13.9 mg/L (IL17i) to 17.6 ± 33 mg/L (TNFi, Table 1).

In psoriasis patients exposed to these different treatments, the risks of all-cause mortality and cardiovascular disease were also considerably different. Whilst in psoriasis exposed to IL17i, no death occurred within the two-year follow-up, this amounted to 0.91% where apremilast use was indicated. Regarding MACE, the highest risks were observed in EHRs where patients were exposed to apremilast (5.14%) or to classic antipsoriatic drugs (8.49%). By contrast, the risk of MACE was lower in all groups exposed to any biologic antipsoriatic treatment. More specifically, 3.74% (TNFi), 3.34% (IL23i, excluding ustekinumab), 2.99% (IL17i), and 2.09% for IL23i (Table 2). Based on disparities in MACE risk between biologic and non-biologic antipsoriatic treatments, a comparative analysis between these was conducted. This aimed to succinctly decipher the cardiovascular outcomes associated with these. The risk of other cardiovascular diseases, which were additionally investigated, is also presented in Table 2.

**Table 2:** Risk of all-cause mortality and cardiovascular disease development in psoriasis patients treated with any of the indicated medications between one day after until two years after index event.

Outcome/Treatment	TNFi	IL17i	IL23i	IL23i, excluding ustekinumab	Apremilast	Classic
All-cause mortality	0.10	0.00	0.41	0.00	0.46	0.32
MACE	1.87	1.50	1.05	1.67	2.57	4.25
Heart failure	0.88	0.75	0.54	1.65	1.57	1.95
Cardiac arrest	0.05	0.74	0.41	1.59	0.46	0.22
Acute myocardial infarction	0.49	0.75	0.41	1.60	0.47	1.30
Cerebral infarction	1.11	0.76	0.41	1.61	1.12	2.39
Hemorrhagic stroke	0.20	0.00	0.40	1.59	0.46	0.46
Peripheral arterial disease	1.95	1.70	1.74	1.66	1.80	4.36
Deep vein thrombosis	0.94	10.82	0.53	1.6	1.03	2.09
Pulmonary embolism	0.41	0.75	0.41	1.58	0.46	1.11

Numbers in table represent proportion of cases per person year. Analysis performed on December 17th, 2023.

### Comparing the risk of all-cause mortality and cardiovascular disease in psoriasis patients exposed to biological as opposed to non-biological systemic antipsoriatic drugs

#### Reduced all-cause mortality risk in psoriasis patients exposed to biologic treatment compared to non-biologic antipsoriatic drugs

**Fig. 2:**
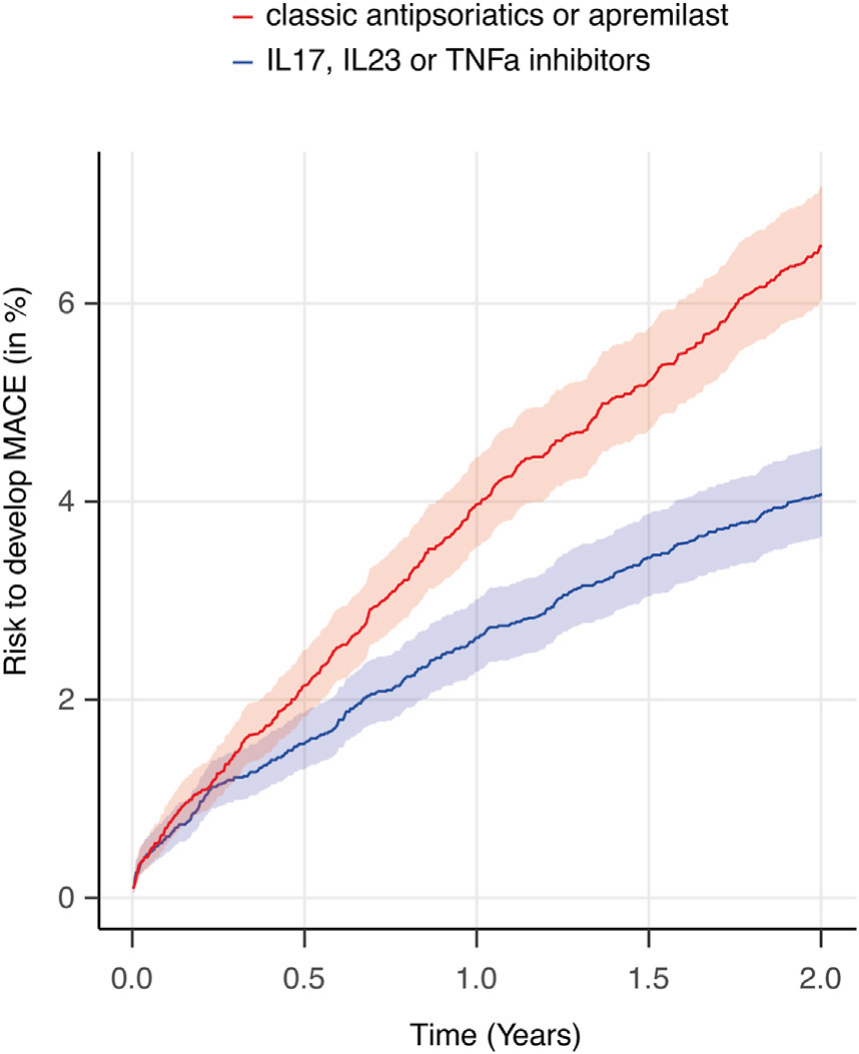
Risk of major adverse cardiac events (MACE) in psoriasis exposed to biologic or non-biologic treatment. EHRs of psoriasis patients exposed to biologic or non-biologic systemic antipsoriatic treatment were retrieved from the TriNetX US Collaborative Research Network. Following propensity-score matching, the risk of MACE was contrasted among the groups. Exposure to the respective drug class was ensured during the two-year follow-up period. In 3.917% of EHRs indicating exposure to biologic antipsoriatic treatment (shown in blue) MACE were documented. In those EHRs with exposure to non-biological treatment (shown in red), this risk amounted to 6.426%, translating into a hazard ratio (solid line) of 1.661 (95% confidence interval [shaded area] 1.432–1.925, p < 0.0001).

**Fig. 3:**
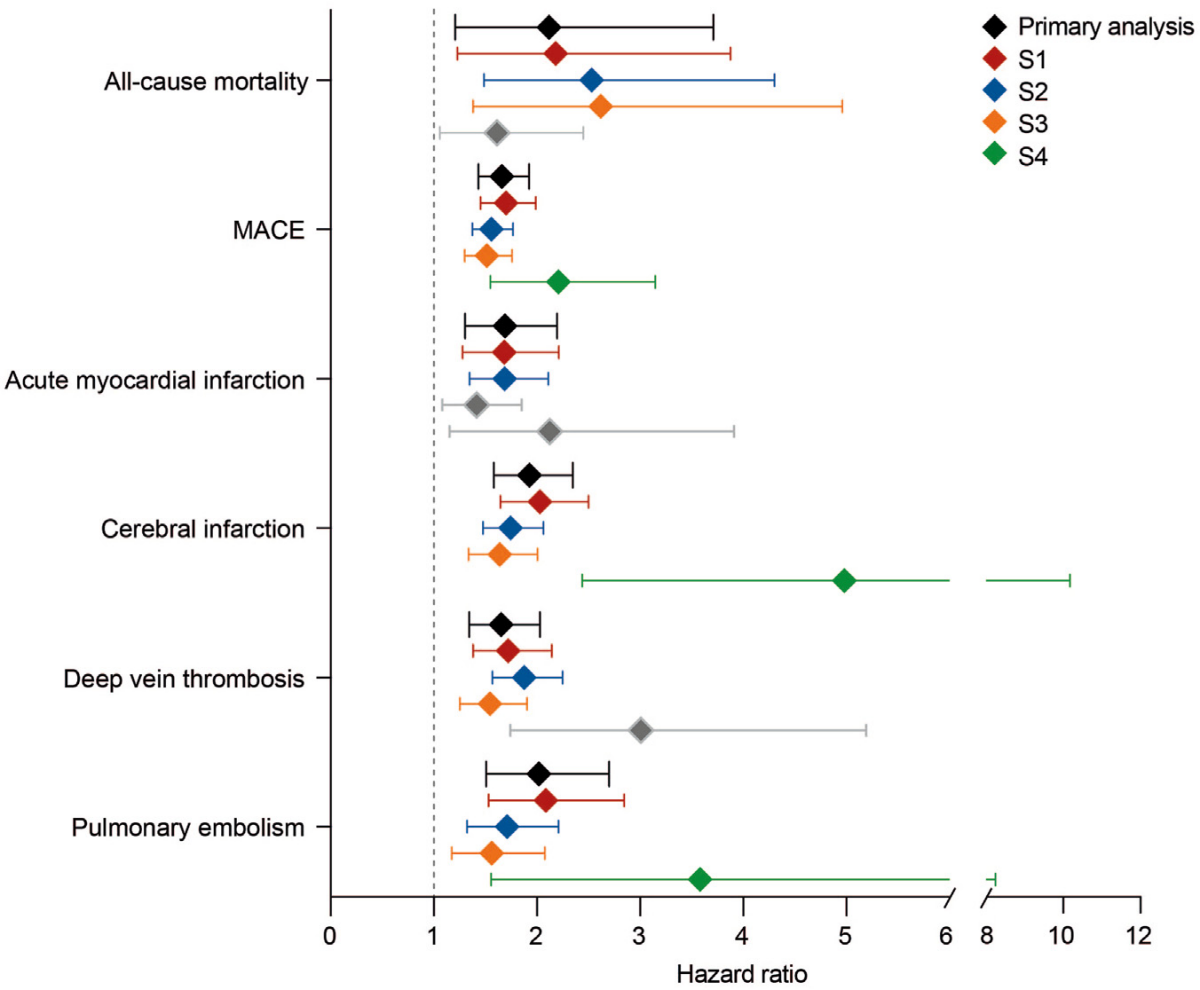
Risk of developing the indicated outcomes in psoriasis patients exposed to biologic or non-biologic systemic antipsoriatic treatment. EHRs of psoriasis patients exposed to biologic or non-biologic systemic antipsoriatic treatment were retrieved from the TriNetX US Collaborative Research Network. Following propensity score matching, the risks of the indicated outcomes was analyzed. In the primary analysis (black) exposure to the respective drug class was ensured for the duration of the two-year follow-up. In the first sensitivity analysis (dark red), outcomes within one month following the index event were excluded to mitigate the potential inclusion of prevalent cases of cardiovascular diseases. In the second sensitivity analysis (blue), a more lenient definition of psoriasis was applied to retrieve a larger number of EHRs. In the third sensitivity analysis (orange), follow-up was restricted to one year with ensured drug exposure during that time. In the fourth sensitivity analysis (green), outcomes prior to the index event were excluded at data retrival and follow up was for 5 years to allow for a better matching of the cohorts. Diamonds represent hazard ratios and error bars correspond to the 95% confidence intervals. Diamonds and error bars shown in gray indicate non-significant results. Please note that the confidence intervals displayed are at the 95% level. Due to the application of Bonferroni adjustment α(adjust) is 0.008. The adjusted confidence interval would be 99.2%.

Over the two-year follow-up period, all-cause mortality was observed in 0.23% of patients undergoing biological treatment, while an over 2-fold higher incidence of 0.49% was documented in those exposed to non-biological treatment. This translates into a hazard ratio (HR) of 2.21 (95% confidence interval [CI] 1.21–3.71, p = 0.0073, Fig. 2, Table 3, Supplement Table S1). This finding persisted in all three sensitivity analyses: In the first sensitivity analysis, excluding outcomes within one month following the index event to mitigate the potential inclusion of prevalent cases of cardiovascular diseases, non-biological treatment was associated with a higher risk of death (HR, 2.18, 95% CI 1.23–3.88, p = 0.0063, Supplement Tables S1 and S2). For the second sensitivity analysis, a more lenient definition of psoriasis was applied to retrieve a larger number of EHRs. Consistent with the primary comparative analysis, outcomes within one day to two years after the index event were considered, and the results were reaffirmed (Supplement Tables S3 and S4). In the third sensitivity analysis, the stringent definition of psoriasis was reinstated, and EHRs with one year of documented use of the specific treatment were included. Outcomes within one day to one year after the index event were considered, and once again, the results of the primary comparative analysis were corroborated (Supplement Tables S5 and S6). The fourth sensitivity analysis excluded any of the outcomes prior to PSM and followed the patients for five years. Mortality risk showed a similar trend to the previous analyses. However, after correction for multiple testing, increased risks for all-cause mortality were not statistically significant (Fig. 3, Supplement Tables S7 and S8).

**Table 3:** Risk of all-cause mortality and cardiovascular disease development in psoriasis patients treated with any of the indicated medications between one day after until two years after index event.

Outcome	Classic or apremilast (cases)			Biologics (Controls)			Analysis					
	N of eligible participants	N of out-comes	Cases per person year, %	N of eligible participants	N of out-comes	Cases per person year, %	Risk difference, %	(95% confidence interval)	Hazard ratio	(95% confidence interval)	p value (α_adj._ = 0.008)	Chi-square
All-cause mortality	7686	38	0.25	7699	18	0.12	0.261	(0.07,0.451)	2.117	(1.208,3.709)	0.0073	0.387
MACE	7127	458	3.21	7301	286	1.96	2.509	(1.786,3.232)	1.661	(1.432,1.925)	<0.0001	0.734
Acute myocardial infarction	7564	151	1.00	7594	90	0.59	0.811	(0.413,1.209)	1.69	(1.302,2.194)	<0.0001	1.714
Cerebral infarction	7444	287	1.93	7538	152	1.01	1.839	(1.299,2.379)	1.928	(1.583,2.346)	<0.0001	2.815
Deep vein thrombosis	7416	237	1.60	7508	146	0.97	1.251	(0.743,1.759)	1.652	(1.344,2.03)	<0.0001	3.163
Pulmonary embolism	7598	136	0.90	7634	68	0.45	0.899	(0.534,1.264)	2.017	(1.508,2.699)	<0.0001	1.819

Analysis performed on December 19th, 2023. Sample sizes vary across the investigated outcomes because outcomes prior to the index event are excluded, and propensity score matching is performed for each analysis. To allow insights into the sample sizes of individual drugs to the groups of drug categories (classic and apremilast as opposed to biologics), we retrieved the probability to be prescribed any of the drugs starting from the documentation of psoriasis until sex weeks later. The probability in ascending order for drug prescription within that time frame were: 2.55% for methotrexate, 2.46% for adalimumab, 2.24% for apremilast, 1.13% for secukinumab, 1.05% for ustekinumab, 1.03% for risankizumab, 1.00% for ixekizumab, 0.63% for etanercept, 0.60% for golimumab, 0.60% for guselkumab, 0.52% for cyclosporine, 0.56% for acitretin, 0.24% for infliximab, 0.1% for certolizumab, 0.05% for tildrakizumab, and 0.02% for brodalumab, and 0.02% for dimethyl fumarate. Assuming treatment with a single compound, 15.37% of psoriasis patients presenting the Health Care Organizations receive any form of systemic treatment. Amongst these, the majority are treated with methotrexate, methotrexate or apremilast—approximately 16% of systemically treated psoriasis patients are managed with either one of these three drugs.

#### Reduced cardiovascular disease risk in psoriasis patients exposed to biologic treatment compared to non-biologic antipsoriatic drugs

Subsequently, the cardiovascular disease risk among psoriasis patients receiving different treatments was conducted. Consistent with the all-cause mortality findings, individuals exposed to non-biologic treatment exhibited a higher risk of MACE at 6.45%, compared to those with biological treatment, where the risk was 3.92%, over the two-year follow-up period. The increased risk amounted to a HR of 1.66 (95% CI 1.43–1.93, p < 0.0001, Figs. 2 and 3, Table 3, Supplement Table S1). This finding persisted in all four sensitivity analyses (Supplement Tables S1–S6). In addition to MACE, all four investigated cardiovascular diseases also demonstrated with an increased risk when exposed to non-biological as opposed to treatment. These included, acute myocardial infarction (HR 1.69, 95% CI 1.30–2.19, p < 0.0001), cerebral infarction (HR 1.93, 95% CI 1.58–2.35, p < 0.0001), as well as deep vein thrombosis (HR 1.65, 95% CI 1.34–2.03, p < 0.0001) and pulmonary embolism (HR 2.02, 95% CI 1.51–2.70, p < 0.0001, Fig. 3, Table 3). Comparing non-biologic treatment to biologic antipsoriatic drugs, the increased risks of MACE, cerebral infarction, and pulmonary embolism persisted across all sensitivity analyses. The increased risk of deep vein thrombosis was consistent in three out of four sensitivity analyses, while the risk of acute myocardial infarction persisted in two out of four sensitivity analyses (Fig. 3, Supplement Tables S2, S4, S6, S8).

### Comparing the risk of death and cardiovascular disease in psoriasis patients exposed to different biological drugs

In the descriptive analysis, notable disparities in the risk of all-cause mortality and cardiovascular disease were evident among psoriasis patients exposed to various biologic treatments. Concurrently, significant differences were observed in potential confounding factors influencing these risks, including the prevalence of essential hypertension, chronic kidney disease, and diabetes mellitus (Tables 1 and 2). To account for this potential bias, we compared the risks of death and cardiovascular disease among TNFi, IL17i and IL23i (including the IL12/23i ustekinumab). With very few exceptions, specifically, a higher risk for cerebral infarction in TNFi-as opposed to IL23i-exposed psoriasis patients, no differences were evident when contrasting the risk of death and cardiovascular disease amongst psoriasis patients exposed to different biologic treatments (Supplement Tables S9–S14).

## Discussion

In this large-scale retrospective cohort study of patients with psoriasis the risk of all-cause mortality and cardiovascular disease was mitigated in patients exposed to biologic treatment, compared with those treated with classic antipsoriatic drugs or apremilast. No difference in all-cause mortality and cardiovascular disease risk was noted when comparing patients treated with any biologic. The results persisted across three sensitivity analyses that investigated longer exclusion periods for prevalent cases, a more lenient psoriasis definition, or a shorter follow-up time.

Life expectancy is reduced in psoriasis patients and cardiovascular events are among the most common causes of death in this cohort.^35^ Hence selection of treatments that modify this risk is of central clinical importance. The data from retrospective cohort studies on this subject, however, remains inconclusive. Some studies reported a benefit of biologic treatment, others observed reduced cardiovascular events in psoriasis patients treated with methotrexate, whilst others noted no impact of treatment on cardiovascular disease or mortality risk. Foremost, studies with biomarker endpoints offer evidence that biological treatment has the potential to improve clinical cardiovascular endpoints: In patients with moderate to severe psoriasis receiving either biologics or topical and/or phototherapy, coronary inflammation -non-invasively assessed by mapping spatial changes of perivascular fat composition-significantly decreased in those treated with biologics, as opposed to the topically or phototherapy group.^36^ This is in line with previous studies that demonstrated a decrease in intima–media thickness under biologic treatment of psoriasis—albeit this was also observed in patients treated with methotrexate.^37^, ^38^, ^39^ In line, randomized controlled trials demonstrated that IL12/23i reduce aortic vascular inflammation, while TNFi and phototherapy reduce CRP and IL-6 serum concentration in patients with psoriasis.^40^ These findings of a potential protective effect for cardiovascular disease risk of biological therapy in psoriasis are supported by observations made in pre-clinical studies.^41^^,^^42^ A decreased risk for all-cause mortality and/or cardiovascular disease was noted in the following retrospective cohort studies: In a retrospective study involving 17,729 patients with psoriasis exposed to TNFi or methotrexate treatment, a lower risk of stroke, transient ischemic attack, and unstable angina was observed in the TNFi-exposed group compared to methotrexate.^43^ Another study demonstrated that systemic anti-inflammatory treatment with biologics or methotrexate was associated with lower cardiovascular disease risk compared to patients treated with other anti-psoriatic therapies, including retinoids, cyclosporine, phototherapy and/or climate therapy.^44^ By contrast in a longitudinal nationwide cohort study conducted in Denmark, methotrexate, but not biologics, cyclosporine, or retinoids, was associated with significantly lower rates of cardiovascular events. In terms of all-cause mortality, patients treated with methotrexate or biologic drugs exhibited lower death rates compared to those under cyclosporine or retinoid therapy.^21^ Contrastingly, the following studies have reported no discernible impact of systemic treatment on the risk of mortality and/or cardiovascular disease: In the Psoriasis Longitudinal Assessment Registry, the risk of MACE was compared between psoriasis patients treated with biologics or topical/phototherapy. Among a total of 7550 patients, 52 MACE events were reported, and the incidence rates were found to be similar across all unmatched treatment groups.^45^ Likewise, a metanalysis of 38 randomized clinical trials showed no significant difference in MACE risk in psoriasis patients exposed to biologic treatment.^24^ These findings were later affirmed in a prospective cohort study^46^ and a metanalysis.^47^ In contrast to afore-mentioned investigations, another study documented a significantly greater risk for MACE with psoriasis patients exposed to IL-12/23i or IL-17i compared to TNFi.^48^

It is noteworthy that only a few of the studies implemented matching for cardiovascular risk factors or affirmed prolonged drug exposure. This, at least in part, may explain the high variability observed in the results. Propensity matching for key risk factors of cardiovascular disease has been implemented herein, addressing the potential bias associated with unequal cardiovascular risk distribution and thereby enhancing internal validity.^49^ The consistency observed between the results of the primary analysis and all sensitivity analyses validates the credibility of the observed decrease in the risk for death and cardiovascular disease in psoriasis patients treated with biologics compared to those under non-biologic treatment.

The interpretation of this study results necessitates consideration of the following limitations: Diagnoses, treatments, and outcomes rely on respective coding, posing a potential source for mis- or non-coding. For instance, this could potentially underestimate the risk of death across all cohorts, as outcomes are only recorded when documented at the HCOs or obtained through NLP. The results are also vulnerable to indication bias. Specifically, the decision to prescribe a biologic for psoriasis is likely influenced by several crucial unmeasured confounders, such as the severity of psoriasis, comorbidity, and economic constraints. These factors might not be fully captured in EHRs or could involve other undocumented elements affecting the choices made by physicians or patients.^50^^,^^51^ The construction of cohorts assumed sustained exposure to the initially prescribed drug for two or more years, based on documentation of the same medication at the index event and at any time within the subsequent two years. Following the same logic, exposure to any other drug under investigation during this period was used as an exclusion criterion. While this approach enhances the likelihood of continued exposure to the initially prescribed drug and excludes use of any other systemic antipsoriatic, it does not preclude the possibility of intermittent pauses or the prescription of another drug within the timeframe spanning one day to two years after the index event. Furthermore, these stringent in- and exclusion criteria lead to relatively small group sizes, Hence, studies over a longer period of time or repetition of the study at a later time point (which will increase the sample size because the database continuously expands) are needed for more accurate assessment. The impact of small molecules, such as tyrosine kinase 2 inhibitors, could not be investigated due to the current absence of a sufficient number of EHRs with follow-up for two years in the database. For PSM, for each patient in each cohort, outcomes of interest and values for each covariate are computed. These data form a matrix of covariate values for each patient, representing the characteristics and measurements relevant to the study. Once these matrices are constructed, logistic regression on the pooled matrices is performed to predict which cohort each patient originates from. This logistic regression model leverages the covariate values to determine the likelihood that a patient belongs to a specific cohort. If covariates are missing, they cannot be considered for PSM and are thus matched with other patients who also lack this information. This may impose a bias. However, this affects all groups systematically. Finally, our findings do not permit the inference of causality, which should be established through prospective clinical trials, (meta)analysis of observational cohorts, and/or completed clinical trials. Notwithstanding these limitations, this large-scale retrospective cohort study on psoriasis patients found a diminished risk of death and cardiovascular disease in those treated with biologics compared to non-biologics. Further validation through prospective clinical trials, meta (analysis) of observational cohorts, or pooled assessments of previous randomized trials, is needed for validation. In the interim, the current findings endorse the use of biologics over non-biologics as a favorable first-line therapy for psoriasis.

## Contributors

Conceived and designed the analysis: RJL; collected the data: KK, MAL, BG, HZ; contributed data or analysis tools: KB, AV; performed the analysis: KK, ELM, DT, RJL; wrote the paper: All authors.

## Data sharing statement

All data shown is provided in the tables or supplement tables.

## Declaration of interests

Diamant Thaci has received honoraria or fees for serving on advisory boards, as a speaker, as a consultant from AbbVie, Amgen, Almirall, Beiersdorf, Bristol-Meiers-Squibb, Boehringer Ingelheim, Galapagos, Leo Pharma, Merck Sharp & Dohme, Morphosys, Lilly, Novartis, Janssen-Cilag, Pfizer, Regeneron, Sanofi, Hexal, Sun Pharmaceuticals, and UCB and grants from Leo Pharma and Novartis. Gema Hernandez is an employee of TriNetX. Henner Zirpel has received support for attending meetings and/or travel from Pfizer, UCB Pharma, Almirall, Janssen, TriNetX. Ralf J. Ludwig has received honoraria for speaking or consulting or has obtained research grants from Monasterium Laboratories, Novartis, Lilly, Bayer, Dompe, Synthon, Argen-X, TriNetX, and Incyte during the last 3 years. All other authors declare no conflict of interest.
