# Supplementary material for: Biological, as opposed to classic antipsoriatic drug or apremilast, treatment mitigates the risk of death and cardiovascular disease in psoriasis
Source: eBioMedicine. 2024 Dec 6;111:105485. doi: 10.1016/j.ebiom.2024.105485 (PMC11665659; doi:10.1016/j.ebiom.2024.105485)
Supplement: Supplement [file mmc1.docx]

| **Characteristics / treatment** | **Before propensity matching** | | | | **After propensity matching** | | | |
| --- | --- | --- | --- | --- | --- | --- | --- | --- |
|  | **Classic or apremilast**  **(Cases)** | **Biologics**  **(Controls)** | **p** | **Std Diff** | **Classic or apremilast**  **(Cases)** | **Biologics**  **(Controls)** | **p** | **Std Diff** |
| N | 10,251 | 13,409 | - | - | 7,726 | 7,726 | n.s. | - |
| **Demographics** | | |  |  |  |  |  |  |
| Age, years  Mean ± SD | 56.3 ± 16 | 45.6 ± 15.9 | < 0.0001 | 0.6705 | 52.6 ± 15.9 | 52.1 ± 14.9 | 0.0448 | 0.0323 |
| Sex, female (%) | 65.693 | 50.59 | < 0.0001 | 0.3098 | 59.449 | 61.468 | 0.0103 | 0.0413 |
| White (%) | 82.305 | 82.626 | 0.5212 | 0.0084 | 32.384 | 82.63 | 0.6874 | 0.0065 |
| **Family history and comorbidity** | | |  |  |  |  |  |  |
| Family history of ischemic heart disease and other diseases of the circulatory system (%) | 6.246 | 3.57 | < 0.0001 | 0.1241 | 4.906 | 4.918 | 0.9703 | 0.0006 |
| Nicotine dependence (%) | 8.941 | 7.723 | 0.0008 | 0.0441 | 8.543 | 8.206 | 0.4502 | 0.0121 |
| Personal history of nicotine dependence (%) | 9.413 | 6.237 | < 0.0001 | 0.1185 | 8.025 | 8.064 | 0.9293 | 0.0014 |
| Essential (primary) hypertension (%) | 32.724 | 20.249 | < 0.0001 | 0.2856 | 27.064 | 27.53 | 0.5156 | 0.0105 |
| Disorders of lipoprotein metabolism and other lipidemias (%) | 28.396 | 17.06 | < 0.0001 | 0.2730 | 22.845 | 23.285 | 0.5161 | 0.0104 |
| Chronic lower respiratory diseases (%) | 15.983 | 10.42 | < 0.0001 | 0.1863 | 13.176 | 13.112 | 0.9052 | 0.0108 |
| Chronic kidney disease (%) | 6.128 | 3.682 | < 0.0001 | 0.1649 | 4.944 | 4.983 | 0.9115 | 0.0019 |
| Neoplasms (%) | 27.501 | 15.589 | < 0.0001 | 0.1134 | 20.878 | 21.965 | 0.0995 | 0.0018 |
| Diabetes mellitus (%) | 14.321 | 9.531 | < 0.0001 | 0.2928 | 12.697 | 12.309 | 0.4656 | 0.0265 |
| **Vitals and laboratory** | | |  |  |  |  |  |  |
| Body mass index, kg/m^2^ (mean [standard deviation]) | 29.9 ± 7.15 | 29.8 ± 7.62 | 0.8636 | 0.0124 | 29.9 ± 7.34 | 30.8 ± 7.41 | 0.0001 | 0.0063 |
| C reactive protein, mg/L (mean [standard deviation]) | 13.8 ± 27.4 | 15.8 ± 30.5 | 0.0148 | 0.1482 | 14.4 ± 28.5 | 14.6 ± 30.3 | 0.8406 | 0.0117 |

**Supplement Table 1.** Demographics, comorbidity and laboratory results of the study population in the US Collaborative Network considering any outcome between one day (or one month) after until two years after index event comparing biologics versus classic antipsoriatic drugs / apremilast. Analysis performed on December 19^th^, 2023. *Abbreviations:* ***Std Diff****: Standardized difference.*

| **Outcome** | **Classic or apremilast (cases)** | | | **Biologics (Controls)** | | | **Analysis** | | | | | |
| --- | --- | --- | --- | --- | --- | --- | --- | --- | --- | --- | --- | --- |
|  | **N of eligible participants** | **N of Out-comes** | **Cases per person year, %** | **N of eligible participants** | **N of Out-comes** | **Cases per person year, %** | **Risk difference, %** | **(95% confidence interval)** | **Hazard ratio** | **(95% confidence interval)** | **Chi-square** | **P value**  **(α_adj._= 0.008)** |
| All-cause mortality | 7,685 | 37 | 0.251 | 7,698 | 17 | 0.115 | 0.261 | (0.074,0.448) | 2.183 | (1.229,3.876) | 0.213 | 0.0063 |
| MACE | 7,078 | 409 | 3.,014 | 7,265 | 250 | 1.795 | 2.337 | (1.651,3.024) | 1.699 | (1.452,1.988) | 0.202 | < 0.0001 |
| Acute myocardial infarction | 7,550 | 137 | 0.947 | 7,586 | 82 | 0.564 | 0.734 | (0.353,1.114) | 1.683 | (1.28,2.213) | 2.442 | 0.0002 |
| Cerebral infarction | 7,419 | 262 | 1.842 | 7,518 | 132 | 0.916 | 1.776 | (1.261,2.29) | 2.028 | (1.645,2.5) | 1.063 | < 0.0001 |
| Deep vein thrombosis | 7,392 | 213 | 1.503 | 7,488 | 126 | 0.878 | 1.199 | (0.719,1.679) | 1.721 | (1.381,2.145) | 1.917 | < 0.0001 |
| Pulmonary embolism | 7,584 | 122 | 0.839 | 7,625 | 59 | 0.404 | 0.835 | (0.49,1.18) | 2.086 | (1.529,2.847) | 1.565 | < 0.0001 |

**Supplement Table 2.** Risk of all-cause mortality and cardiovascular disease development in psoriasis patients treated with any of the indicated medications between one month after until two years after index event. Cases per person year were thus calculated by dividing by 1.9167. Sample sizes vary across the investigated outcomes because outcomes prior to the index event are excluded, and propensity score matching is performed for each analysis. Analysis performed on December 19^th^, 2023.

| **Characteristics / treatment** | **Before propensity matching** | | | | **After propensity matching** | | | |
| --- | --- | --- | --- | --- | --- | --- | --- | --- |
|  | **Classic or apremilast**  **(Cases)** | **Biologics**  **(Controls)** | **p** | **Std Diff** | **Classic or apremilast**  **(Cases)** | **Biologics**  **(Controls)** | **p** | **Std Diff** |
| N | 14,470 | 18,929 | - | - | 10,892 | 10,892 | - | - |
| **Demographics** | | |  |  |  |  |  |  |
| Age, years  Mean ± SD | 56.5 ± 15.7 | 45.8 ± 15.8 | < 0.0001 | 0.6785 | 52.8 ± 15.5 | 52.4 ± 14.8 | 0.0412 | 0.0277 |
| Sex, female (%) | 63.19 | 49.002 | < 0.0001 | 0.2889 | 57.051 | 58.942 | 0.0047 | 0.0383 |
| White (%) | 79.938 | 80.693 | 0.0864 | 0.0190 | 80.297 | 80.04 | 0.6342 | 0.0064 |
| **Family history and comorbidity** | | |  |  |  |  |  |  |
| Family history of ischemic heart disease and other diseases of the circulatory system (%) | 5.55 | 3.308 | < 0.0001 | 0.1092 | 4.297 | 4.324 | 0.9203 | 0.0014 |
| Nicotine dependence (%) | 9.332 | 7.748 | < 0.0001 | 0.0567 | 8.566 | 8.538 | 0.9421 | 0.0010 |
| Personal history of nicotine dependence (%) | 8.821 | 5.806 | < 0.0001 | 0.1160 | 7.51 | 7.336 | 0.6234 | 0.0067 |
| Essential (primary) hypertension (%) | 33.21 | 20.804 | < 0.0001 | 0.2822 | 27.699 | 28.039 | 0.5761 | 0.0076 |
| Disorders of lipoprotein metabolism and other lipidemias (%) | 29.295 | 17.92 | < 0.0001 | 0.2703 | 23.586 | 24.247 | 0.2528 | 0.0155 |
| Chronic lower respiratory diseases (%) | 16.21 | 10.479 | < 0.0001 | 0.1691 | 13.111 | 13.478 | 0.4247 | 0.0108 |
| Chronic kidney disease (%) | 6.074 | 3.705 | < 0.0001 | 0.1101 | 5.123 | 5.004 | 0.6879 | 0.0054 |
| Neoplasms (%) | 29.114 | 16.772 | < 0.0001 | 0.2967 | 22.787 | 23.311 | 0.3591 | 0.0124 |
| Diabetes mellitus (%) | 14.455 | 9.839 | < 0.0001 | 0.1417 | 12.743 | 12.725 | 0.9676 | 0.0006 |
| **Vitals and laboratory** | | |  |  |  |  |  |  |
| Body mass index, kg/m^2^ (mean [standard deviation]) | 29.9 ± 7.08 | 30 ± 7.45 | 0.7044 | 0.0080 | 26.79 | 26.928 | < 0.0001 | 0.1140 |
| C reactive protein, mg/L (mean [standard deviation]) | 14.2 ± 28.9 | 15.7 ± 30.8 | 0.0301 | 0.0515 | 14.2 ± 27.6 | 14.4 ± 30.1 | 0.7594 | 0.0089 |

**Supplement Table 3.** Demographics, comorbidity and laboratory results of the study population in the US Collaborative Network considering any outcome between one day after until two years after index event comparing biologics versus classic antipsoriatic drugs / apremilast, including all EHRs with ICD10:L40. Analysis performed on December 19^th^, 2023. *Abbreviations:* ***Std Diff****: Standardized difference.*

| **Outcome** | **Classic or apremilast (cases)** | | | **Biologics (Controls)** | | | **Analysis** | | | | | |
| --- | --- | --- | --- | --- | --- | --- | --- | --- | --- | --- | --- | --- |
|  | **N of eligible participants** | **N of Out-comes** | **Cases per person year, %** | **N of eligible participants** | **N of Out-comes** | **Cases per person year, %** | **Risk difference, %** | **(95% confidence interval)** | **Hazard ratio** | **(95% confidence interval)** | **Chi-square** | **P value**  **(α_adj._= 0.008)** |
| All-cause mortality | 10,846 | 48 | 0.222 | 10,852 | 19 | 0.088 | 0.267 | (0.12,0.415) | 1,488 | (1.488,4.304) | 1.365 | 0.0004 |
| MACE | 10,068 | 610 | 3.030 | 10,310 | 405 | 1.969 | 2.131 | (1.532,2.729) | 1.559 | (1.375,1.768) | 0.015 | < 0.0001 |
| Acute myocardial infarction | 10,672 | 204 | 0.956 | 10,724 | 122 | 0.569 | 0.774 | (0.446,1.102) | 1.687 | (1.348,2.111) | 0.059 | < 0.0001 |
| Cerebral infarction | 10,522 | 370 | 1.758 | 10,644 | 216 | 1.015 | 1.487 | (1.045,1.929) | 1.745 | (1.475,2.063) | 1.640 | < 0.0001 |
| Deep vein thrombosis | 10,469 | 335 | 1.600 | 10,608 | 182 | 0.858 | 1.484 | (1.066,1.902) | 1.877 | (1.567,2.248) | 3.309 | < 0.0001 |
| Pulmonary embolism | 10,728 | 158 | 0.737 | 10,766 | 93 | 0.432 | 0.609 | (0.322,0.896) | 1.71 | (1.323,2.209) | 0.261 | < 0.0001 |

**Supplement Table 4.** Risk of all-cause mortality and cardiovascular disease development in psoriasis patients treated with any of the indicated medications between one day after until two years after index event, including all EHRs with ICD10:L40. Sample sizes vary across the investigated outcomes because outcomes prior to the index event are excluded, and propensity score matching is performed for each analysis. Analysis performed on December 19^th^, 2023.

| **Characteristics / treatment** | **Before propensity matching** | | | | **After propensity matching** | | | |
| --- | --- | --- | --- | --- | --- | --- | --- | --- |
|  | **Classic or apremilast**  **(Cases)** | **Biologics**  **(Controls)** | **p** | **Std Diff** | **Classic or apremilast**  **(Cases)** | **Biologics**  **(Controls)** | **p** | **Std Diff** |
| N | 14,207 | 11,021 | - | - | 18,444 | 11,021 | - | - |
| **Demographics** | | |  |  |  |  |  |  |
| Age, years  Mean ± SD | 56.1 ± 16.3 | 45.9 ± 16.1 | < 0.0001 | 0.6251 | 52.5 ± 16.1 | 52.1 ± 15.3 | 0.0750 | 0.0240 |
| Sex, female (%) | 64,86 | 51.65 | < 0.0001 | 0.2703 | 59.477 | 61.02 | 0.0193 | 0.0315 |
| White (%) | 81.544 | 81.43 | 0.7919 | 0.0030 | 81.889 | 81.853 | 0.9442 | 0.0009 |
| **Family history and comorbidity** | | |  |  |  |  |  |  |
| Family history of ischemic heart disease and other diseases of the circulatory system (%) | 6.865 | 4.012 | < 0.0001 | 0.1261 | 5.335 | 5.263 | 0.8099 | 0.0032 |
| Nicotine dependence (%) | 9.961 | 8.398 | < 0.0001 | 0.0542 | 9.482 | 9.21 | 0.4875 | 0.0094 |
| Personal history of nicotine dependence (%) | 10.556 | 7.23 | < 0.0001 | 0.1170 | 9.083 | 8.847 | 0.5399 | 0.0083 |
| Essential (primary) hypertension (%) | 33.085 | 21.333 | < 0.0001 | 0.2664 | 27.42 | 28.155 | 0.2232 | 0.0164 |
| Disorders of lipoprotein metabolism and other lipidemias (%) | 29.253 | 18.12 | < 0.0001 | 0.2641 | 23.764 | 24.526 | 0.1862 | 0.0178 |
| Chronic lower respiratory diseases (%) | 16.954 | 10.938 | < 0.0001 | 0.1743 | 13.991 | 13.946 | 0.9226 | 0.0013 |
| Chronic kidney disease (%) | 6.745 | 4.017 | < 0.0001 | 0.1211 | 5.381 | 5.344 | 0.9048 | 0.0016 |
| Neoplasms (%) | 28.282 | 16.426 | < 0.0001 | 0.2875 | 21.858 | 22.602 | 0.1841 | 0.0179 |
| Diabetes mellitus (%) | 14.998 | 10.221 | < 0.0001 | 0.1443 | 13.021 | 13.284 | 0.5633 | 0.0078 |
| **Vitals and laboratory** | | |  |  |  |  |  |  |
| Body mass index, kg/m^2^ (mean [standard deviation]) | 30 ± 7.27 | 30.1 ± 7.63 | 0.3860 | 0.3860 | 29.9 ± 7.4 | 30.8 ± 7.45 | < 0.0001 | 0.1174 |
| C reactive protein, mg/L (mean [standard deviation]) | 14.4 ± 29.7 | 15.4 ± 31.4 | 0.1637 | 0.0328 | 14.5 ± 30.3 | 14.3 ± 31 | 0.7653 | 0.0085 |

**Supplement Table 5.** Demographics, comorbidity and laboratory results of the study population in the US Collaborative Network considering any outcome between one day after until one year after index event comparing biologics versus classic antipsoriatic drugs / apremilast. Note, that due to the inclusion criteria for continued treatment with the respective drugs was 12 months, as opposed to 24 months in the previous analyses. Analysis performed on December 19^th^, 2023. *Abbreviations:* ***Std Diff****: Standardized difference.*

| **Outcome** | **Classic or apremilast (cases)** | | | **Biologics (Controls)** | | | **Analysis** | | | | | |
| --- | --- | --- | --- | --- | --- | --- | --- | --- | --- | --- | --- | --- |
|  | **N of eligible participants** | **N of Out-comes** | **Cases per person year, %** | **N of eligible participants** | **N of Out-comes** | **Cases per person year, %** | **Risk difference, %** | **(95% confidence interval)** | **Hazard ratio** | **(95% confidence interval)** | **Chi-square** | **P value**  **(α_adj._= 0.008)** |
| All-cause mortality | 10,972 | 34 | 0.31 | 10,976 | 13 | 0.118 | 0.191 | (0.069,0.314) | 2.619 | (1.382,4.962) | 0.027 | 0.0022 |
| MACE | 10,098 | 418 | 4.139 | 10,306 | 284 | 2.756 | 1.384 | (0.883,1.885) | 1.512 | (1.301,1.758) | 0.324 | < 0.0001 |
| Acute myocardial infarction | 10,762 | 128 | 1.189 | 10,809 | 91 | 0.842 | 0.347 | (0.08,0.615) | 1.415 | (1.082,1.852) | 0.017 | 0.0109 |
| Cerebral infarction | 10,573 | 245 | 2.317 | 10,705 | 152 | 1.42 | 0.897 | (0.533,1.261) | 1.639 | (1.338,2.006) | 0.661 | < 0.0001 |
| Deep vein thrombosis | 10,529 | 223 | 2.118 | 10,682 | 147 | 1.376 | 0.742 | (0.389,1.095) | 1.545 | (1.254,1.902) | 0.079 | < 0.0001 |
| Pulmonary embolism | 10,820 | 121 | 1.118 | 10,864 | 78 | 0.718 | 0.4 | (0.146,0.654) | 1.561 | (1.174,2.075) | 0.358 | 0.0020 |

**Supplement Table 6.** Risk of all-cause mortality and cardiovascular disease development in psoriasis patients treated with any of the indicated medications between one day after until one year after index event. Sample sizes vary across the investigated outcomes because outcomes prior to the index event are excluded, and propensity score matching is performed for each analysis. Note, that due to the inclusion criteria for continued treatment with the respective drugs was 12 months, as opposed to 24 months in the previous analyses. Analysis performed on December 19^th^, 2023.

| **Characteristics / treatment** | **Before propensity matching** | | | | **After propensity matching** | | | |
| --- | --- | --- | --- | --- | --- | --- | --- | --- |
|  | **Classic or apremilast**  **(Cases)** | **Biologics**  **(Controls)** | **p** | **Std Diff** | **Classic or apremilast**  **(Cases)** | **Biologics**  **(Controls)** | **p** | **Std Diff** |
| N | 6,240 | 11,896 | - | - | 5,375 | 5,375 | - | - |
| **Demographics** | | |  |  |  |  |  |  |
| Age, years  Mean ± SD | 54 ± 16.2 | 43.6 ± 15.6 | < 0.0001 | 0.6528 | 51.5 ± 15.8 | 51.3 ± 14.9 | 0.4280 | 0.0153 |
| Sex, female (%) | 64.343 | 49.496 | < 0.0001 | 0.3033 | 60.316 | 60.856 | 0.5671 | 0.0110 |
| White (%) | 81.282 | 81.103 | 0.7694 | 0.0046 | 81.358 | 81.991 | 0.3966 | 0.0164 |
| **Family history and comorbidity** | | |  |  |  |  |  |  |
| Family history of ischemic heart disease and other diseases of the circulatory system (%) | 3.237 | 1.967 | < 0.0001 | 0.0798 | 2.791 | 2.809 | 0.9534 | 0.0011 |
| Nicotine dependence (%) | 6.058 | 5.943 | 0.7574 | 0.0048 | 6.307 | 5.414 | 0.0487 | 0.0380 |
| Personal history of nicotine dependence (%) | 5.401 | 4.27 | 0.0006 | 0.0527 | 5.321 | 4.726 | 0.1577 | 0.0273 |
| Essential (primary) hypertension (%) | 23.349 | 15.19 | < 0.0001 | 0.2080 | 20.874 | 21.228 | 0.6531 | 0.0087 |
| Disorders of lipoprotein metabolism and other lipidemias (%) | 21.635 | 13.458 | < 0.0001 | 0.2162 | 18.735 | 18.716 | 0.9803 | 0.0005 |
| Chronic lower respiratory diseases (%) | 11.779 | 7.818 | < 0.0001 | 0.1335 | 10.214 | 10.195 | 0.9746 | 0.0006 |
| Chronic kidney disease (%) | 2.244 | 1.471 | 0.0002 | 0.0572 | 1.953 | 2.028 | 0.7824 | 0.0053 |
| Neoplasms (%) | 23.253 | 13.542 | < 0.0001 | 0.2526 | 19.665 | 20.633 | 0.2112 | 0.0241 |
| Diabetes mellitus (%) | 9.391 | 6.532 | < 0.0001 | 0.1058 | 8.763 | 8.242 | 0.3329 | 0.0187 |
| **Vitals and laboratory** | | |  |  |  |  |  |  |
| Body mass index, kg/m^2^ (mean [standard deviation]) | 30.5 ± 7.5 | 29.9 ± 7.89 | 0.0018 | 0.0764 | 30.6 ± 7.52 | 30.7 ± 7.52 | 0.6992 | 0.0116 |
| C reactive protein, mg/L (mean [standard deviation]) | 11.9 ± 23.4 | 14.9 ± 26.9 | 0.0004 | 0.1228 | 12.2 ± 23.8 | 13.6 ± 25.9 | 0.1744 | 0.0573 |

**Supplement Table 7.** Demographics, comorbidity and laboratory results of the study population in the US Collaborative Network considering any outcome between one day after until five years after index event comparing biologics versus classic antipsoriatic drugs / apremilast. Retrieval of EHRs excluded those with any of the outcomes documented at or any time before the index event, as opposed to excluding outcomes after the propensity score matching. Retrieval performed on July 1^st^, 2024. *Abbreviations:* ***Std Diff****: Standardized difference.*

| **Outcome** | **Classic or apremilast (cases)** | | | **Biologics (Controls)** | | | **Analysis** | | | | | |
| --- | --- | --- | --- | --- | --- | --- | --- | --- | --- | --- | --- | --- |
|  | **N of eligible participants** | **N of Out-comes** | **Cases per person year, %** | **N of eligible participants** | **N of Out-comes** | **Cases per person year, %** | **Risk difference, %** | **(95% confidence interval)** | **Hazard ratio** | **(95% confidence interval)** | **Chi-square** | **P value**  **(α_adj._= 0.008)** |
| All-cause mortality | 5,250 | 59 | 0.225 | 5,250 | 35 | 0.134 | 0.457 | (0.097,0.817) | 1.613 | (1.061,2.45) | 0.165 | 0.0238 |
| MACE | 5,242 | 100 | 0.382 | 5,245 | 44 | 0.168 | 1.069 | (0.624,1.514) | 2.207 | (1.548,3.147) | 0.587 | < 0.0001 |
| Acute myocardial infarction | 5,250 | 33 | 0.126 | 5,247 | 15 | 0.057 | 0.343 | (0.085,0.601) | 2.124 | (1.153,3.91) | 0.47 | 0.0133 |
| Cerebral infarction | 5,247 | 46 | 0.176 | 5,249 | 10 | 0.038 | 0.686 | (0.408,0.965) | 4.983 | (2.439,10.18) | 0.169 | < 0.0001 |
| Deep vein thrombosis | 5,249 | 10 | 0.037 | 5,410 | 10 | 0.037 | 0 | (-0.162,0.162) | 5.002 | (0.584,42.812) | 0.052 | 0.1024 |
| Pulmonary embolism | 5,248 | 26 | 0.099 | 5,249 | 10 | 0.038 | 0.305 | (0.081,0.529) | 3.581 | (1.554,8.25) | 0.113 | 0.0014 |

**Supplement Table 8.** Risk of all-cause mortality and cardiovascular disease development in psoriasis patients treated with any of the indicated medications between one day after until five years after index event. Retrieval of EHRs excluded those with any of the outcomes documented at or any time before the index event, as opposed to excluding outcomes after the propensity score matching. Sample sizes vary across the investigated outcomes because outcomes prior to the index event are excluded, and propensity score matching is performed for each analysis. Analysis performed on July 1^st^, 2024.

| **Characteristics / treatment** | **Before propensity matching** | | | | **After propensity matching** | | | |
| --- | --- | --- | --- | --- | --- | --- | --- | --- |
|  | **TNFi**  **(Cases)** | **IL23i**  **(Controls)** | **p** | **Std Diff** | **TNFi**  **(Cases)** | **IL23i**  **(Controls)** | **p** | **Std Diff** |
| N | 6,518 | 1,247 | - | - | 1,245 | 1,245 | - | - |
| **Demographics** | | |  |  |  |  |  |  |
| Age, years  Mean ± SD | 46 ± 16.3 | 46.1 ± 15.7 | 0.7644 | 0.0094 | 44.7 ± 16.1 | 46.1 ± 15.7 | 0.0278 | 0.0882 |
| Sex, female (%) | 48.538 | 48.395 | 0.9260 | 0.0029 | 48,916 | 48.353 | 0.7790 | 0.0112 |
| White (%) | 82.954 | 80.257 | 0.0217 | 0.0697 | 78.876 | 80.321 | 0.3707 | 0.0359 |
| **Family history and comorbidity** | | |  |  |  |  |  |  |
| Family history of ischemic heart disease and other diseases of the circulatory system (%) | 3.415 | 3.21 | 0.7137 | 0.0115 | 3.373 | 3.213 | 0.8223 | 0.0090 |
| Nicotine dependence (%) | 6.846 | 8.026 | 0.1359 | 0.0450 | 8.273 | 8.032 | 0.8261 | 0.0088 |
| Personal history of nicotine dependence (%) | 5.969 | 6.581 | 0.4073 | 0.0252 | 6.265 | 6.586 | 0.7437 | 0.0131 |
| Essential (primary) hypertension (%) | 19.415 | 20.626 | 0.3242 | 0.0303 | 18.635 | 20.562 | 0.2257 | 0.0486 |
| Disorders of lipoprotein metabolism and other lipidemias (%) | 16.338 | 18.299 | 0.0889 | 0.0518 | 16.948 | 18.233 | 0.3997 | 0.0338 |
| Chronic lower respiratory diseases (%) | 9.831 | 10.433 | 0.5146 | 0.0200 | 9.157 | 10.442 | 0.2808 | 0.0432 |
| Chronic kidney disease (%) | 3.769 | 4.655 | 0.1396 | 0.0441 | 3.855 | 4.578 | 0.3695 | 0.0360 |
| Neoplasms (%) | 14.431 | 17.817 | 0.0022 | 0.0922 | 17.108 | 17.751 | 0.6726 | 0.0169 |
| Diabetes mellitus (%) | 8.662 | 9.47 | 0.3557 | 0.0282 | 7.791 | 9.398 | 0.1527 | 0.0573 |
| **Vitals and laboratory** | | |  |  |  |  |  |  |
| Body mass index, kg/m^2^ (mean [standard deviation]) | 28.8 ± 7.29 | 30.7 ± 7.19 | < 0.0001 | 0.2600 | 28.6 ± 6.62 | 30.6 ± 7.13 | 0.0006 | 0.3005 |
| C reactive protein, mg/L (mean [standard deviation]) | 17.6 ± 33 | 10.6 ± 16.4 | 0.0070 | 0.2673 | 15.5 ± 27.6 | 10.6 ± 16.4 | 0.0496 | 0.2146 |

**Supplement Table 9.** Demographics, comorbidity and laboratory results of the study population in the US Collaborative Network considering any outcome between one day after until two years after index event comparing TNFi versus IL23i. Analysis performed on December 19^th^, 2023. *Abbreviations:* ***Std Diff****: Standardized difference.*

| **Outcome** | **TNFi (Cases)** | | | **IL23i (Controls)** | | | **Analysis** | | | | | |
| --- | --- | --- | --- | --- | --- | --- | --- | --- | --- | --- | --- | --- |
|  | **N of eligible participants** | **N of Out-comes** | **Cases per person year, %** | **N of eligible participants** | **N of Out-comes** | **Cases per person year, %** | **Risk difference, %** | **(95% confidence interval)** | **Hazard ratio** | **(95% confidence interval)** | **Chi-square** | **P value**  **(α_adj._= 0.008)** |
| All-cause mortality | 1,243 | 10 | 0.403 | 1,241 | 10 | 0.403 | -0.001 | (-0.704,0.702) | 1.998 | (0.181,22.039) | 2.248 | 0.5642 |
| MACE | 1,195 | 41 | 1.716 | 1,200 | 25 | 1.042 | 1.348 | (0.037,2.658) | 1.662 | (1.011,2.733) | 1.442 | 0.0430 |
| Acute myocardial infarction | 1,231 | 11 | 0.447 | 1,231 | 10 | 0.406 | 0.081 | (-0.645,0.808) | 1.575 | (0.611,4.064) | 0.953 | 0.3431 |
| Cerebral infarction | 1,220 | 29 | 1.189 | 1,232 | 10 | 0.406 | 1.565 | (0.575,2.556) | 2.95 | (1.438,6.053) | 0.103 | 0.0020 |
| Deep vein thrombosis | 1,215 | 24 | 0.988 | 1,228 | 13 | 0.530 | 0.917 | (-0.053,1.886) | 1.872 | (0.953,3.677) | 0.468 | 0.0641 |
| Pulmonary embolism | 1,234 | 10 | 0.405 | 1,237 | 10 | 0.404 | 0.002 | (-0.705,0.709) | 1.002 | (0.398,2.523) | 0.595 | 0.9972 |

**Supplement Table 10.** Risk of all-cause mortality and cardiovascular disease development in psoriasis patients one day after until two years after index event comparing TNFi versus IL23i. Sample sizes vary across the investigated outcomes because outcomes prior to the index event are excluded, and propensity score matching is performed for each analysis. Analysis performed on December 19^th^, 2023. Not significant results are shown in grey.

| **Characteristics / treatment** | **Before propensity matching** | | | | **After propensity matching** | | | |
| --- | --- | --- | --- | --- | --- | --- | --- | --- |
|  | **IL17i**  **(Cases)** | **IL23i**  **(Controls)** | **p** | **Std Diff** | **TNFi**  **(Cases)** | **IL23i**  **(Controls)** | **p** | **Std Diff** |
| N | 682 | 1,247 | - | - | 668 | 668 | - | - |
| **Demographics** | | |  |  |  |  |  |  |
| Age, years  Mean ± SD | 48.4 ± 15 | 46.1 ± 15.7 | 0.0021 | 0.1477 | 48.1 ± 15 | 48.3 ± 14.7 | 0.8123 | 0.0130 |
| Sex, female (%) | 51.173 | 48.395 | 0.2434 | 0.0556 | 51.198 | 52.246 | 0.7015 | 0.0210 |
| White (%) | 80.938 | 80.257 | 0.7180 | 0.0172 | 80.689 | 80.09 | 0.7828 | 0.0151 |
| **Family history and comorbidity** | | |  |  |  |  |  |  |
| Family history of ischemic heart disease and other diseases of the circulatory system (%) | 5.425 | 3.21 | 0.0176 | 0.1091 | 4.641 | 4.042 | 0.5913 | 0.0294 |
| Nicotine dependence (%) | 8.651 | 8.026 | 0.6332 | 0.0226 | 8.383 | 6.886 | 0.3029 | 0.0564 |
| Personal history of nicotine dependence (%) | 6.598 | 6.581 | 0.9884 | 0.0007 | 6.587 | 5.988 | 0.6521 | 0.0247 |
| Essential (primary) hypertension (%) | 25.953 | 20.626 | 0.0074 | 0.1263 | 24.701 | 22.156 | 0.2722 | 0.0601 |
| Disorders of lipoprotein metabolism and other lipidemias (%) | 19.648 | 18.299 | 0.4681 | 0.0344 | 19.012 | 15.12 | 0.0587 | 0.1036 |
| Chronic lower respiratory diseases (%) | 9.238 | 10.433 | 0.4029 | 0.0402 | 9.431 | 7.934 | 0.3312 | 0.0532 |
| Chronic kidney disease (%) | 3.079 | 4.655 | 0.0952 | 0.0818 | 3.144 | 2.695 |  | 0.6259 |
| Neoplasms (%) | 17.009 | 17.817 | 0.6554 | 0.0213 | 17.066 | 15.269 | 0.3725 | 0.0488 |
| Diabetes mellitus (%) | 13.49 | 9.47 | 0.0068 | 0.1263 | 12.126 | 10.778 | 0.4394 | 0.0423 |
| **Vitals and laboratory** | | |  |  |  |  |  |  |
| Body mass index, kg/m^2^ (mean [standard deviation]) | 31.6 ± 7.67 | 30.7 ± 7.19 | 0.2094 | 0.1201 | 31.5 ± 7.69 | 30.6 ± 7.59 | 0.3247 | 0.1099 |
| C reactive protein, mg/L (mean [standard deviation]) | 8.87 ± 13.9 | 10.6 ± 16.4 | 0.3620 | 0.1125 | 8.76 ± 13.7 | 9.68 ± 15 | 0.6553 | 0.0635 |

**Supplement Table 11.** Demographics, comorbidity and laboratory results of the study population in the US Collaborative Network considering any outcome between one day after until two years after index event comparing IL17i versus IL23i. Sample sizes vary across the investigated outcomes because outcomes prior to the index event are excluded, and propensity score matching is performed for each analysis. Analysis performed on December 20^th^, 2023. *Abbreviations:* ***Std Diff****: Standardized difference.*

| **Outcome** | **IL17i (Cases)** | | | **IL23i (Controls)** | | | **Analysis** | | | | | |
| --- | --- | --- | --- | --- | --- | --- | --- | --- | --- | --- | --- | --- |
|  | **N of eligible participants** | **N of Out-comes** | **Cases per person year, %** | **N of eligible participants** | **N of Out-comes** | **Cases per person year, %** | **Risk difference, %** | **(95% confidence interval)** | **Hazard ratio** | **(95% confidence interval)** | **Chi-square** | **P value**  **(α_adj._= 0.008)** |
| All-cause mortality | 665 | 0 | 0.000 | 666 | 10 | 0.751 | -1.502 | (-2.425,-0.578) | -* | -* | 0.998 | 0.3177 |
| MACE | 625 | 17 | 1.360 | 643 | 11 | 0.856 | 1.009 | (-0.613,2.631) | 1.593 | (0.746,3.402) | 1.476 | 0.2244 |
| Acute myocardial infarction | 653 | 10 | 0.766 | 662 | 10 | 0.756 | 0.021 | (-1.302,1.344) | 7.124 | (0.876,57.902) | 4.606 | 0.0319 |
| Cerebral infarction | 652 | 10 | 0.767 | 662 | 10 | 0.756 | 0.023 | (-1.301,1.347) | 1.355 | (0.47,3.904) | 0.318 | 0.5726 |
| Deep vein thrombosis | 659 | 10 | 0.759 | 660 | 10 | 0.758 | 0.002 | (-1.317,1.321) | 1 | (0.397,2.519) | 0.000 | 0.9999 |
| Pulmonary embolism | 659 | 10 | 0.759 | 664 | 10 | 0.753 | 0.011 | (-1.304,1.326) | 0.805 | (0.216,2.999) | 0.105 | 0.7463 |

**Supplement Table 12.** Risk of all-cause mortality and cardiovascular disease development in psoriasis patients one day after until two years after index event comparing IL17i versus IL23i. Sample sizes vary across the investigated outcomes because outcomes prior to the index event are excluded, and propensity score matching is performed for each analysis. Analysis performed on December 20^th^, 2023. *cannot be calculated because 0 events in one of the outcomes.

| **Characteristics / treatment** | **Before propensity matching** | | | | **After propensity matching** | | | |
| --- | --- | --- | --- | --- | --- | --- | --- | --- |
|  | **IL17i**  **(Cases)** | **TNFi**  **(Controls)** | **p** | **Std Diff** | **IL17i**  **(Cases)** | **TNFi**  **(Controls)** | **p** | **Std Diff** |
| N | 692 | 6,502 | - | - | 690 | 690 | - | - |
| **Demographics** | | |  |  |  |  |  |  |
| Age, years  Mean ± SD | 48.4 ± 15.1 | 45.9 ± 16.3 | < 0.0001 | 0.1608 | 48.4 ± 15 | 47.1 ± 15.9 | 0.1377 | 0.0800 |
| Sex, female (%) | 51.156 | 48.596 | 0.2003 | 0.0512 | 51.159 | 54.638 | 0.1956 | 0.0697 |
| White (%) | 81.069 | 32.891 | 0.2283 | 0.0474 | 81.014 | 80 | 0.6343 | 0.0256 |
| **Family history and comorbidity** | | |  |  |  |  |  |  |
| Family history of ischemic heart disease and other diseases of the circulatory system (%) | 5.636 | 3.425 | 0.0032 | 0.1065 | 5.362 | 5.217 | 0.9043 | 0.0065 |
| Nicotine dependence (%) | 9.393 | 3.425 | 0.0032 | 0.0848 | 5.362 | 5.217 | 0.9043 | 0.0468 |
| Personal history of nicotine dependence (%) | 6.936 | 6.048 | 0.3543 | 0.0361 | 6.957 | 6.377 | 0.6660 | 0.0232 |
| Essential (primary) hypertension (%) | 26.59 | 19.716 | < 0.0001 | 0.1635 | 26.377 | 27.246 | 0.7154 | 0.0196 |
| Disorders of lipoprotein metabolism and other lipidemias (%) | 20.809 | 16.646 | 0.0056 | 0.1069 | 20.725 | 17.246 | 0.0995 | 0.0888 |
| Chronic lower respiratory diseases (%) | 3.035 | 3.826 | 0.2976 |  | 3.043 | 2.319 | 0.4047 |  |
| Chronic kidney disease (%) | 3.035 | 3.826 | 0.2976 | 0.0435 | 3.043 | 2.319 | 0.4047 | 0.0449 |
| Neoplasms (%) | 18.353 | 15.273 | 0.0337 | 0.0824 | 18.261 | 16.552 | 0.3941 | 0.0459 |
| Diabetes mellitus (%) | 13.728 | 8.778 | < 0.0001 | 0.1571 | 13.478 | 12.029 | 0.4197 | 0.0435 |
| **Vitals and laboratory** | | |  |  |  |  |  |  |
| Body mass index, kg/m^2^ (mean [standard deviation]) | 31.7 ± 7.66 | 28.8 ± 7.27 | < 0.0001 | 0.3871 | 31.7 ± 7.68 | 28.7 ± 7.25 | 0.0002 | 0.3949 |
| C reactive protein, mg/L (mean [standard deviation]) | 10.5 ± 22 | 17.2 ± 32.4 | 0.0272 | 0.2399 | 10.5 ± 22 | 20.8 ± 43 | 0.0215 | 0.3003 |

**Supplement Table 13.** Demographics, comorbidity and laboratory results of the study population in the US Collaborative Network considering any outcome between one day after until two years after index event comparing IL17i versus TNFi. Sample sizes vary across the investigated outcomes because outcomes prior to the index event are excluded, and propensity score matching is performed for each analysis. Analysis performed on January 2^nd^, 2024.

| **Outcome** | **IL17i (Cases)** | | | **TNFi (Controls)** | | | **Analysis** | | | | | |
| --- | --- | --- | --- | --- | --- | --- | --- | --- | --- | --- | --- | --- |
|  | **N of eligible participants** | **N of Out-comes** | **Cases per person year, %** | **N of eligible participants** | **N of Out-comes** | **Cases per person year, %** | **Risk difference, %** | **(95% confidence interval)** | **Hazard ratio** | **(95% confidence interval)** | **Chi-square** | **P value**  **(α_adj._= 0.008)** |
| All-cause mortality | 687 | 0 | 0,000 | 688 | 10^1^ | 0.727 | -1.453 | (-2.348,-0.559) | -* | -* | -* | 0.0833 |
| MACE | 646 | 17 | 1,316 | 652 | 23 | 1.764 | -0.896 | (-2.775,0.983) | 0.741 | (0.396,1.387) | 0.620 | 0.3472 |
| Acute myocardial infarction | 675 | 10 | 0,741 | 680 | 10 | 0.736 | 0.011 | (-1.273,1.295) | 1.175 | (0.395,3.497) | 0.200 | 0.7713 |
| Cerebral infarction | 673 | 10 | 0,743 | 674 | 14 | 1.039 | -0.591 | (-2.004,0.821) | 0.571 | (0.239,1.36) | 0.383 | 0.1996 |
| Deep vein thrombosis | 680 | 10 | 0,736 | 679 | 12 | 0.884 | -0.297 | (-1.639,1.045) | 0.831 | (0.359,1.924) | 0.000 | 0.6653 |
| Pulmonary embolism | 680 | 10 | 0,736 | 680 | 10 | 0.736 | 0 | (-1.28,1.28) | 0.714 | (0.227,2.25) | 0.587 | 0.5634 |

**Supplement Table 14.** Risk of all-cause mortality and cardiovascular disease development in psoriasis patients one day after until two years after index event comparing IL17i versus TNFi. Sample sizes vary across the investigated outcomes because outcomes prior to the index event are excluded, and propensity score matching is performed for each analysis. Analysis performed on December 20^th^, 2023. ^1^By default 1-10 outcomes are expressed as 10 outcomes. *cannot be calculated because 0 events in one of the outcomes. Analysis performed on January 2^nd^, 2024.
